# Genetic pleiotropy between mood disorders, metabolic, and endocrine traits in a multigenerational pedigree

**DOI:** 10.1038/s41398-018-0226-3

**Published:** 2018-10-12

**Authors:** Rachel L. Kember, Liping Hou, Xiao Ji, Lars H. Andersen, Arpita Ghorai, Lisa N. Estrella, Laura Almasy, Francis J. McMahon, Christopher Brown, Maja Bućan

**Affiliations:** 1 Department of Genetics, Perelman School of Medicine, University of Pennsylvania, Philadelphia, PA 19104 USA; 2 Human Genetics Branch, National Institute of Mental Health Intramural Research Program, National Institutes of Health, Bethesda, MD 20892 USA; 3 Genomics and Computational Biology Program, Perelman School of Medicine, University of Pennsylvania, Philadelphia, PA 19104 USA; 4 Lancaster General Health/Penn Medicine, University of Pennsylvania Health System, Lancaster, PA 17602 USA; 5 Department of Biomedical and Health Informatics, Children’s Hospital of Philadelphia, Philadelphia, PA 19104 USA; 6 Department of Psychiatry, Perelman School of Medicine, University of Pennsylvania, Philadelphia, PA 19104 USA

## Abstract

Bipolar disorder (BD) is a mental disorder characterized by alternating periods of depression and mania. Individuals with BD have higher levels of early mortality than the general population, and a substantial proportion of this is due to increased risk for comorbid diseases. To identify the molecular events that underlie BD and related medical comorbidities, we generated imputed whole-genome sequence data using a population-specific reference panel for an extended multigenerational Old Order Amish pedigree (*n* = 394), segregating BD and related disorders. First, we investigated all putative disease-causing variants at known Mendelian disease loci present in this pedigree. Second, we performed genomic profiling using polygenic risk scores (PRS) to establish each individual’s risk for several complex diseases. We identified a set of Mendelian variants that co-occur in individuals with BD more frequently than their unaffected family members, including the R3527Q mutation in *APOB* associated with hypercholesterolemia. Using PRS, we demonstrated that BD individuals from this pedigree were enriched for the same common risk alleles for BD as the general population (β = 0.416, *p* = 6 × 10^−4^). Furthermore, we find evidence for a common genetic etiology between BD risk and polygenic risk for clinical autoimmune thyroid disease (*p* = 1 × 10^−4^), diabetes (*p* = 1 × 10^−3^), and lipid traits such as triglyceride levels (*p* = 3 × 10^−4^) in the pedigree. We identify genomic regions that contribute to the differences between BD individuals and unaffected family members by calculating local genetic risk for independent LD blocks. Our findings provide evidence for the extensive genetic pleiotropy that can drive epidemiological findings of comorbidities between diseases and other complex traits.

## Introduction

Psychiatric disorders frequently co-occur with other medical illnesses, markedly reducing patients’ quality of life. Individuals with mood disorders have higher levels of early mortality than the general population, in part due to comorbid medical disease^1^. In addition, individuals with a higher burden of medical illness display not only a higher occurrence but also an increased severity of psychiatric symptoms^2^. Bipolar disorder (BD) is a highly heritable mood disorder characterized by recurrent periods of depression and mania. Individuals with BD have increased rates of asthma, diabetes, hyperlipidemia, epilepsy, and thyroid disease, among other diseases^3^.

Historically, increased rates of medical illness in patients with psychiatric disorders had been attributed to the side effects of antipsychotic medication or to a reduced ability to maintain a healthy lifestyle. However, recent evidence suggests that shared genetic risk loci or common biological pathways may underlie the pervasive pleiotropy between psychiatric and non-psychiatric disorders^4–7^. Pleiotropy can be identified at the level of individual alleles, or genetic correlations between disorders can be calculated genome wide to quantify the proportion of shared associated loci between traits^5^.

Comorbidity arising from pleiotropic loci has been noted in Mendelian disorders, with a significant number of Mendelian disease-causing variants leading to complex phenotypes^8^. In individual-level data gathered from medical records of over 110 million patients, Mendelian variants were found to contribute non-additively to risk for a subset of complex diseases^9^. Furthermore, common variants associated with complex disease are enriched in Mendelian disease genes^9^. Shared genetic influences between common complex traits have also been identified. Using data from genome-wide association studies (GWAS), several groups have identified loci underlying multiple traits^6,7^. Such cross-phenotype associations^10^ have been found even between distinct traits; for example, a non-synonymous variant in *SLC39A8* is associated with both schizophrenia and height, among others^7^.

Genetic correlations have demonstrated the shared genetic influences between multiple clusters of diseases, including significant correlations between psychiatric disorders^11–14^. Shared genetic etiology has been identified between BD and both schizophrenia and major depressive disorder^11^, suggesting extensive biological pleiotropy between these psychiatric conditions^15^. Given the polygenic nature of these disorders^16^, this result is unsurprising, as polygenicity is consistent with comorbidity and pleiotropy^17^. Gratten et al.^17^ also notes that genetic correlations between BD data sets are more variable, possibly suggesting greater genetic heterogeneity within BD compared with other psychiatric phenotypes.

Population isolates are frequently utilized in genetic studies of disease in order to reduce the genetic and phenotypic heterogeneity found in outbred populations. Disease-gene identification in population isolates usually attempts to identify both common and low-frequency variants observed within and across families. Moreover, founder effects can lead to an increase in allele frequencies for many deleterious alleles and clusters of deleterious variants on shared haplotypes. Genetic studies of the Old Order Amish led to the identification of over 200 Mendelian disease loci^18–20^, and the same population has been extensively utilized in studies of complex metabolic and psychiatric diseases^21–26^.

The Amish Study of Major Affective Disorder (ASMAD) is a large extended pedigree collected over 30 years ago with an initial focus on BD. In addition to a significant enrichment of mood disorders in this family (~150 family members, corresponding to one-third of the pedigree), the pedigree includes individuals with autoimmune thyroid disorder^27^ and Ellis–van Creveld syndrome, an autosomal recessively inherited chondrodysplastic dwarfism^28,29^. Our previous work on this pedigree revealed a complex and polygenic inheritance of BD with multiple linkage regions and clusters of BD risk alleles on different haplotypes, supporting a high degree of locus and allelic heterogeneity^21,22^. Further dissection of the genetic architecture of mental illness in this pedigree has been greatly enhanced by the recently established whole-genome sequence-based imputation reference panel for the Anabaptist population (based on whole-genome sequence for 265 Amish and Mennonite individuals; Anabaptist Genome Reference Panel, AGRP;^30^).

In this study, we used the AGRP in combination with genotypes for the ASMAD pedigree (394 family members) to permit the identification of all known disease-causing and loss-of-function (LoF) variants at known Mendelian loci in subjects with mood disorders. In parallel, we performed genomic profiling using polygenic risk scores (PRS) to establish the individuals’ risk for several complex traits and diseases. Long-range phased haplotypes, estimated using the extended pedigree, permitted the exploration of co-segregation between bipolar risk factors and medical disease loci. We find that a set of 12 Mendelian diseases co-occur in BD individuals more or less frequently than in their unaffected family members, and that risk scores for metabolic and endocrine traits are higher in BD individuals, indicating a common genetic etiology for these traits in the extended Amish family.

## Methods

The genetic–epidemiologic study of BD among the Old Order Amish in Pennsylvania (ASMAD) has been well documented^31,32^ and the sample collection methods have been previously described in detail^21,22^. Genotyping was performed on 394 samples from the extended Amish pedigree using Illumina Omni 2.5 M SNP arrays at the Center for Applied Genomics (Children’s Hospital of Pennsylvania, Philadelphia, PA). Genotypes were phased and imputed with SHAPEIT2^33^ and IMPUTE2^34^, respectively, using the Anabaptist reference panel^30^. In total, 2,379,855 variants were imputed in 394 individuals, of which 1,372,783 variants remained after QC (see Supplemental Methods: Phasing and Imputation). Whole-genome sequencing (WGS) for 80 Old Order Amish family members (including 30 parent–child trios) was performed by Complete Genomics Inc. (CGI; Mountain View, CA) using a sequence-by-ligation method^35^ (see Supplemental Methods: Whole-Genome Sequencing).

Known disease genes and mutations were identified using the Human Genome Mutation Database (HGMD) (see Supplemental Methods: Human disease catalog). Following curation, we classified 22 variants as likely to cause the disease phenotype in the ASMAD family based on their allelic status (see Supplemental Methods: Curation of HGMD disease-causing variants). Using VEP LOFTEE^36^, we identified 1001 high confidence loss-of-function variants (HC-LoF), of which 167 are in disease genes (see Supplemental Methods: Loss-of-function variants).

Association analysis for all variants was performed using EMMAX^37^, and for all genes using MONSTER^38^ (see Supplemental Methods: EMMAX and MONSTER). Association rules, defined using the arules package^39^, were applied to determine whether any Mendelian diseases were comorbid with BD in the ASMAD pedigree (see Supplemental Methods: Association rule discovery). PRS for each individual were generated for multiple traits and diseases using the PRSice package^40^ (see Supplemental Methods: Polygenic risk scores). GWAS summary statistics for all traits were selected based on the sample size (where multiple GWAS for the same trait was available, we selected the one with the largest sample size) and the reference population (European ancestry was selected) (Supplemental Table 9). PRS were standardized and tested for association with the phenotype while accounting for relatedness using linear-mixed model analyses in the pedigreemm package^41^ (see Supplemental Methods: Statistical analysis). In addition, we calculated the extent of polygenic transmission disequilibrium for PRS^42^ between the affected and unaffected siblings within nuclear families (see Supplemental Methods: Polygenic transmission disequilibrium). Finally, drawing on work from Shi et al.^43^, on local genetic correlation, we developed a method for establishing local genetic risk, i.e., genetic risk based on specific regions of the genome (see Supplemental Methods: Local polygenic risk).

## Results

### Identifying the spectrum of mutations in disease genes

By combining the SNP genotype data from 394 ASMAD family members with the Anabaptist Genome Reference Panel^30^, we established imputed whole-genome sequence for the entire ASMAD extended family. Available phenotype records for this collection are limited to information about bipolar and related neuropsychiatric disorders (Supplemental Table 1). However, the availability of imputed genotypes allows genetic profiling of ASMAD family members and identification of carriers of known Mendelian disease variants, although presentation of Mendelian disease in family members could not be confirmed due to restrictions on re-contacting individuals in this legacy collection.

First, we sought to establish all damaging mutations present in ASMAD. We identified 250 variants in 239 genes that are predicted to be deleterious and are common in ASMAD family members (> 5%), but rare in 1000 Genomes and ExAC (< 2%). Disease genes, defined using the Human Genome Mutation Database (HGMD^44^) by selecting those with known disease-causing mutations (HGMD-DM), were enriched among the set of genes containing these variants (57 HGMD-DM out of 239 genes, OR = 1.58, *p* = 3.7 × 10^−3^, two-sided Fisher’s exact test, Supplemental Table 2). We next performed both gene-based and variant-based association tests to identify loci associated with BD in the ASMAD pedigree. Although none were genome-wide significant (Supplemental Figures 1 and 2), HGMD-DM genes are enriched among both sets of genes and sets of variants that are nominally associated with BD in this pedigree. Out of the top 6 genes that have *p*-values < 0.001, five of these are known disease genes (OR = 20.3, *p* = 1 × 10^−3^, two-sided Fisher’s exact test, Supplemental Table 3). Out of the top 44 exonic variants that have *p*-values < 0.001, 17 of these are in known disease genes (OR = 2.2, *p* = 1 × 10^−2^, two-sided Fisher’s exact test, Supplemental Table 4).

In light of these initial results and the previously noted trend toward a higher burden of CNVs in HGMD-DM genes in subjects with BD^22^, we closely examined the variants in disease genes to screen for the presence of Mendelian diseases in the entire pedigree. Out of 3456 HGMD-DM genes, 239 contain either known disease-causing variants or LoF variants in ASMAD. Following extensive curation of variants (see Supplemental Methods: Curation of HGMD disease-causing variants), 62 were identified as high confidence disease-causing, of which 17 are known to be more common in the Amish (> 5% difference compared to 1000 Genomes) (Supplemental Figure 3, Supplemental Table 5). Based on the allelic state of these variants in all individuals, we predict that 22 of these are likely to cause disease due to being present in a homozygous (for recessive diseases) or heterozygous (for dominant diseases) state (Supplemental Table 6). Burden of the genomically predicted medical conditions varies between nuclear families, with some families (and individuals within these families) carrying variants for up to five Mendelian diseases (Supplemental Figures 4–21). Furthermore, each individual has on an average 31 LoF variants in disease genes, with five disease genes predicted to be completely inactivated.

### BD individuals show comorbidity with a specific set of genetically inferred Mendelian diseases

**Fig. 1:**
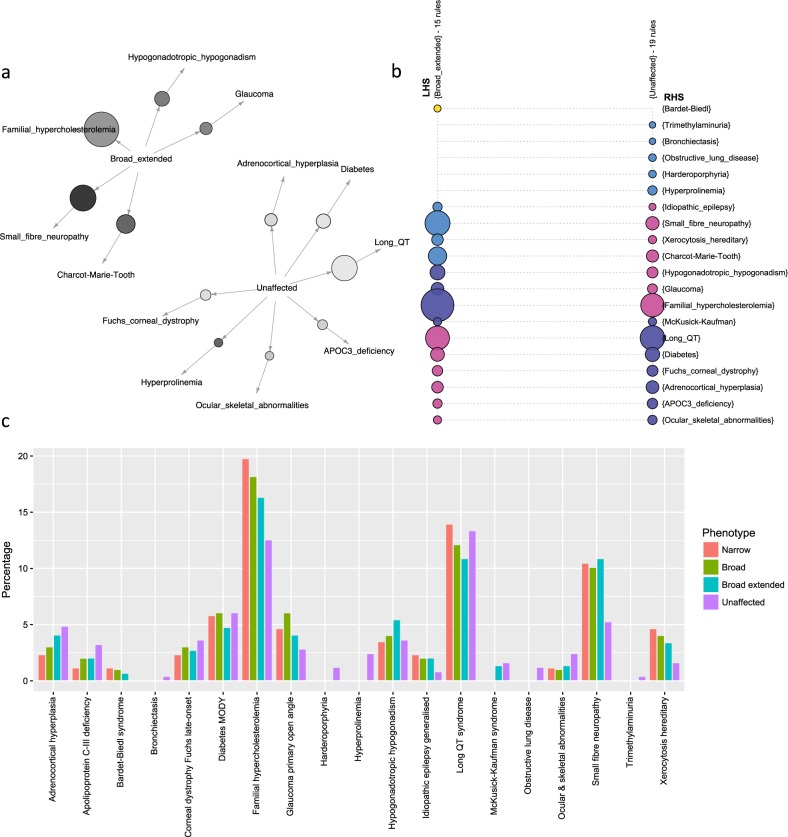
Profile of co-occurring Mendelian diseases varies between affected and unaffected individuals. **a** and **b**: Individuals genetically diagnosed with Mendelian diseases. Association rule discovery looks for combinations of variables that occur together more frequently than expected by chance. Diseases that co-occur more than expected differ between individuals affected with mood disorder and unaffected individuals. **a**: Graph for 12 rules and **b**: grouped matrix for 34 rules with mood disorder-affected status (either “broad extended” or “unaffected”) as the antecedent and Mendelian disease as the consequent. Size of the circle demonstrates confidence in that rule. Color indicates lift (**a**: darker = higher lift, **b**: blue = higher lift, pink = lower lift). **c**: Individuals carrying disease-causing variants. Percentage of narrow, broad, and broad extended individuals (see “common polygenic risk for bipolar disorder” in the Results section for explanation of phenotype) carrying disease-causing variants compared with percentage of unaffected individuals with disease-causing variants. Some variants are found in a higher percentage of affected individuals

Overall burden of disease-causing variants and LoF variants in disease genes is not associated with BD status in the pedigree. To therefore identify if a specific set of inferred Mendelian diseases show comorbidity with BD, we applied association rule discovery to determine the predicted disease status that co-occur more frequently than expected with affected or unaffected status of mood disorders (Fig. 1a, b; rule confidence: 0.015–0.123, rule lift: 1.012–1.694). In the ASMAD pedigree, BD individuals are more likely to carry variants causing familial hypercholesterolemia, small fiber neuropathy, and xerocytosis hereditary (Fig. 1c). Variants causing adrenocortical hyperplasia and apolipoprotein C-III deficiency are found more frequently in unaffected individuals.

Half of the genetically inferred Mendelian diseases (6 out of 12) that were identified by association rule discovery as either enriched or depleted in BD individuals are associated with cardiovascular disease or endocrine traits. As a consequence, we explored additional damaging variants associated with lipid phenotypes (Table 1) and found that they are present at a higher frequency in individuals with BD compared with unaffected individuals, whereas protective lipid variants are found at a lower frequency. We also found that a higher percentage of BD individuals than expected have multiple lipid variants (Supplemental Figure 22). Overall, over one-third (34.0%) of the affected individuals (broad extended phenotype) carry at least one variant associated with damaging lipid or iron overload effects, compared with just under one quarter of unaffected individuals (24.7%).

**Table 1:** Variants associated with lipid and cardiovascular phenotypes

Gene	Variant	rsID	Effect	% Unaffected	% BPI/BPII	% Other psychiatric phenotype
*APOB*	Arg3527Gln	rs5742904	Damaging lipid variant	12.6	21.3	10.3
*ABCG8*	Gly574Arg	rs137852988	Damaging lipid variant	5.7	11.3	12.3
*HFE*	Cys282Tyr	rs1800562	Iron overload	8.9	15.0	8.8
*CYBRD1*	Arg226His	rs62181680	Iron overload	1.2	3.8	4.4
*LPA*	c.4289+1G>A	rs41272114	Cardioprotective	5.3	3.8	1.5
*APOC3*	Arg19Ter	rs76353203	Cardioprotective	3.3	2.5	1.5
*KCNH2*	Gly803Arg	rs199473669	Damaging variant affecting heart rhythm	11.0	11.3	5.9
*SCN5A*	Glu462Lys	rs199473572	Damaging variant affecting heart rhythm	2.8	1.3	0
*SNTA1*	Ala257Gly	rs56157422	Damaging variant affecting heart rhythm	1.2	1.3	0

Damaging variants associated with lipid phenotypes are found at a higher frequency in individuals with BD, including the G574R mutation in *ABCG8* associated with hyperabsorption and sisterolemia, and the R3527Q mutation in *APOB*. In addition to the heterozygotes detected, three homozygotes for the R3527Q mutation were found within the same nuclear family. Protective lipid variants are found at a lower frequency in BD individuals, including a variant in *LPA* associated with a reduction in thermogenic lipoprotein. Variants associated with heart rhythm (in *KCNH2, SCN5A,* and *SNTA1*) show no allele frequency difference between affected and unaffected individuals

### Common polygenic risk for BD

To compare the genetic architecture of BD in the Amish with a non-founder population and to further explore the enrichment of variants associated with lipid traits in BD subjects, we sought to measure the aggregate effect of common variants underlying both psychiatric and endocrine/metabolic traits in family members with and without mood disorders. To achieve this, we evaluated different phenotypic models from the most defined (BPI individuals only) to the most general (individuals with any psychiatric phenotype, including minor psychiatric symptoms). Heritability estimates generated for this pedigree previously have shown significant heritability for mood disorder phenotypes^21^. Using an updated pedigree structure based on accurate genealogical records, we estimated narrow sense heritability for the following phenotype models: narrow (BPI and BPII only), broad (BPI, BPII, and MDDR), broad extended (presence of any psychiatric phenotype), depression (MDDR, MDD, and minor depression), and well. We found significant heritability for all phenotype models (Supplemental Table 7), with broad extended being the most heritable phenotype (h^2^ = 0.81, *p* = 9.76 × 10^−10^).

**Fig. 2:**
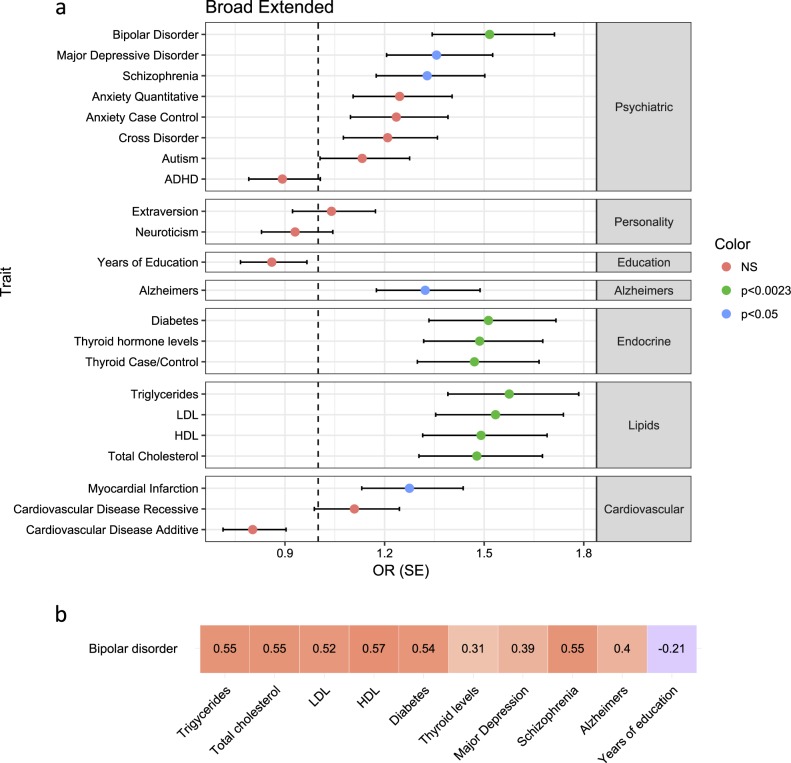
Association of polygenic risk scores for multiple traits with bipolar disorder (broad extended phenotype) in ASMAD. **a** Risk scores for all traits tested are displayed, split by the trait type. Circles indicate the association of the risk scores with mood disorder in the ASMAD pedigree, color of circles shows the level of significance, and error bars show ±standard error. **b** Correlation of risk scores with bipolar disorder risk score within the ASMAD population. Color represents strength and direction of correlation (red – positive, blue – negative). Number in each square is the correlation coefficient (*r*)

**Fig. 3:**
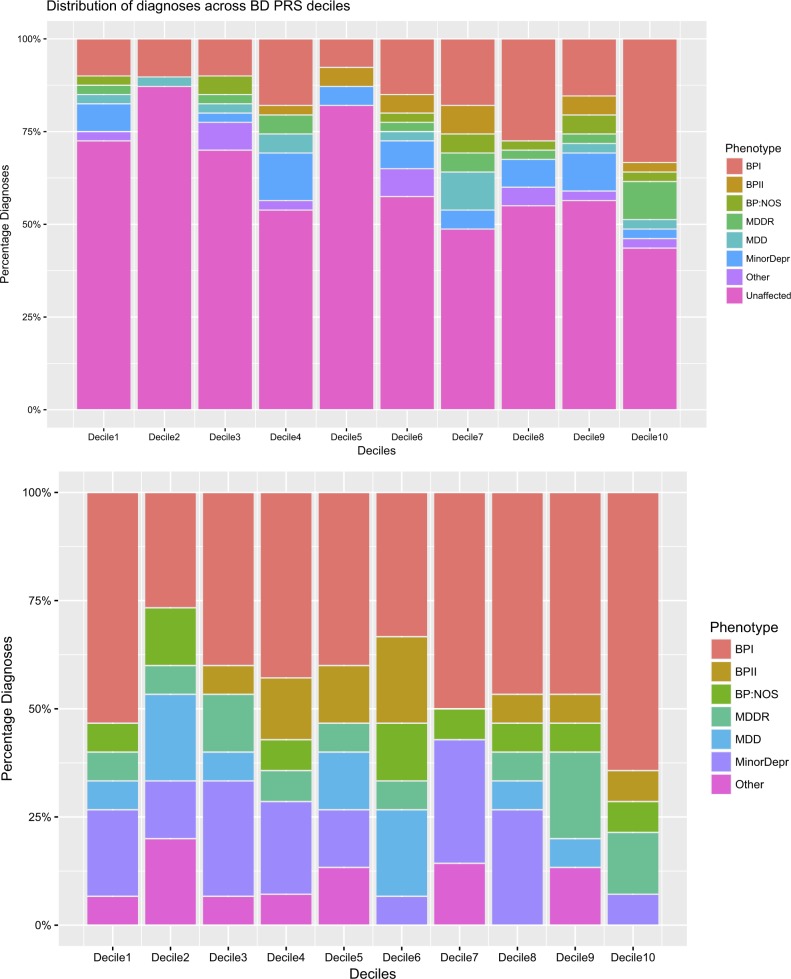
Percentage of individuals with affected status for each decile of polygenic risk score. Top: all individuals, including unaffected individuals, are shown. Bottom: affected individuals only are shown

We generated BD PRS in ASMAD based on a GWAS for BD in over 50,000 individuals of European descent^44^. BD risk score was significantly associated with affected phenotype for broad extended (β = 0.416, SE = 0.121, *p* = 5.9 × 10^−4^, Fig. 2a), broad (β = 0.523, SE = 0.129, *p* = 4.8 × 10^−5^), and narrow phenotypes (β = 0.499, SE = 0.138, *p* = 3 × 10^−4^), but not for depression phenotype (β = 0.273, SE = 0.155, *p* = 7.8 × 10^−2^). To verify this result, we used a modified polygenic transmission disequilibrium test (see Supplemental Methods: Polygenic transmission disequilibrium) and found a statistically significant deviation in risk scores, with affected individuals having a higher polygenic risk than their unaffected siblings (mean deviation 0.27, *p* = 3 × 10^−2^). This increase is mostly driven by higher risk scores of individuals with BPI, BPII, BP:NOS, and MDDR (Supplemental Figure 23). The percentage of affected individuals, on an average, increases with increasing deciles of PRS (Fig. 3a). Furthermore, the percentage of individuals with BPI, BPII, and BP:NOS is the highest in the 10th PRS decile, whereas the percentage of individuals with minor depression or other non-specified disorder is the lowest in this decile (Fig. 3b). Offspring with an affected parent (either one or both parents affected) has higher risk scores than offspring from unaffected parents (Supplemental Figure 24). Descendants that can trace their lineage back to the two pioneer members of this pedigree (“in family”) have significantly higher risk scores for BD than Amish individuals who are “married in” (β = 1.653, SE = 0.370, *p* = 8 × 10^−6^, Supplemental Figure 25), suggesting that the pioneer individuals carried high risk for BD. Risk scores vary across the pedigree, although they are more similar within nuclear families (Supplemental Figure 26). We found that higher inbreeding values (measured as a higher number of observed homozygous genotypes than expected), but not increased average length of homozygous regions, are correlated with an increased risk score for BD (*r* = 0.69, *p* = 2.2 × 10^−15^, Supplemental Figure 27), suggesting a role for increased homozygosity in common disease risk.

### Common risk variants for disease suggest pleiotropy between common complex traits and BD

We generated risk scores for 21 additional traits using GWAS summary data from European populations (see Supplemental Table 9 for full list), including common psychiatric and neurological disorders, anthropometric and social traits, and endocrine and metabolic phenotypes, and tested for an association between the risk score and presence of mood disorder. Risk scores for clinical autoimmune thyroid disease, lipid traits, and type 2 diabetes were found to be significantly associated with BD phenotype (broad extended, broad and narrow) (Fig. 2a), whereas only thyroid hormone, LDL, and triglyceride risk scores were nominally associated with depression phenotype (for full results, see Supplemental table 8). Additionally, risk scores for BD are positively correlated with risk scores for lipid traits (TG *r* = 0.55, *p* < 2.2 × 10^−16^; TC *r* = 0.55, *p* < 2.2 × 10^−16^; LDL *r* = 0.52, *p* < 2.2 × 10^−16^; HDL *r* = 0.57, *p* < 2.2 × 10^−16^, Supplemental Figure 28), diabetes (*r* = 0.54, *p* < 2.2 × 10^−16^), thyroid levels (thyroid hormone levels *r* = 0.31, *p* = 1.6 × 10^−10^), major depression (*r* = 0.39, *p* = 6.4 × 10^−16^), schizophrenia (*r* = 0.55, *p* < 2.2 × 10^−16^), and Alzheimer’s disease (*r* = 0.40, *p* = 2.9 × 10^−16^) and negatively correlated with years of education (*r* = −0.21, *p* = 2 × 10^−5^) (Fig. 2b), suggesting an overlap between loci that contribute to risk for these traits.

To verify that the association between the above risk scores and BD in this pedigree is not driven by the individuals who carry the variants for related Mendelian diseases, we performed a “leave-one-out analysis”. For each of the 22 Mendelian variants predicted to cause disease phenotype in the ASMAD family (Supplemental Table 6), we identified and removed the set of individuals carrying those variants. We then re-calculated the association between genetic risk and BD in the remaining set of individuals. For example, we removed 55 individuals carrying the APOB R3527Q variant for familial hypercholesterolemia and tested for an association between BD and LDL risk score in the remaining set of 339 individuals (β = 0.43, *p* = 0.0006). In all scenarios, the association between the risk score and BD remained statistically significant with a similar effect size to the initial finding (Supplemental table 10). This finding confirms that strong effect disease-causing variants are not driving the association between risk scores based on common variants and BD phenotype.

### Identifying pleiotropic regions of the genome

To establish which loci contribute to the genome-wide risk score differences between affected and unaffected individuals, we calculated the risk scores for each approximately independent LD block (*n* = 1703), as defined by Berisa and Pickrell^61^. This allowed us to partition the genome and analyze the risk for each local region to identify whether (a) a single region or multiple regions are underlying the overall difference in polygenic risk between affected and unaffected family members and (b) if the regions underlying the differences in risk are the same between the different traits and disorders analyzed.

**Fig. 4:**
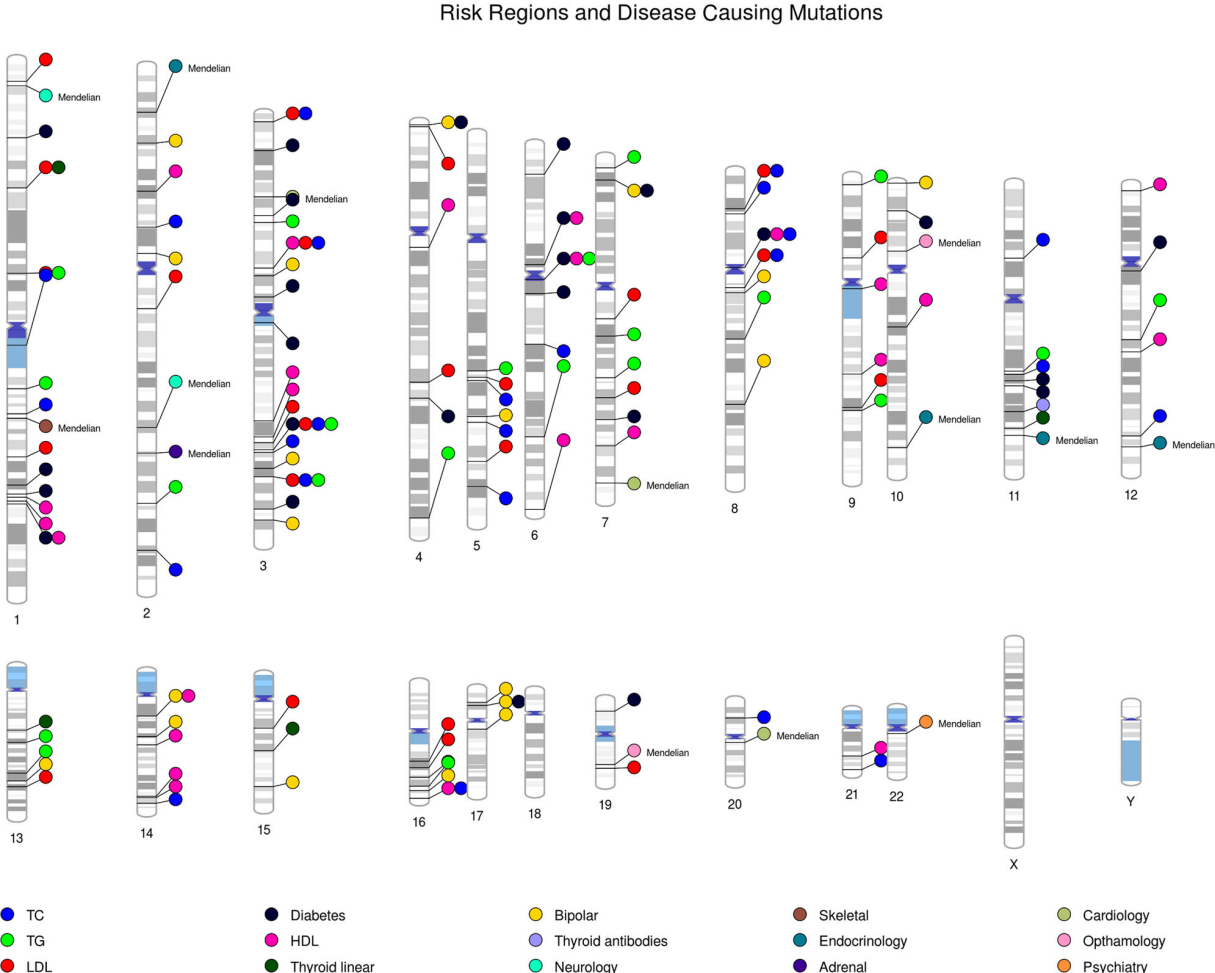
Locations of genomic regions that contribute to the genome-wide risk score differences between the affected and unaffected individuals for bipolar disorder (yellow), diabetes (black), HDL (pink), LDL (red), total cholesterol (blue), triglycerides (light green) thyroid antibodies (lavender), and thyroid hormone (dark green). The location of Mendelian variants which are associated with either affected or unaffected status in this pedigree are also included (denoted by the notation: Mendelian)

We identified a single genomic region on chromosome 15 for BD local polygenic risk (15q25.2-q25.3) that was significantly associated with mood disorder in the ASMAD pedigree (β = 0.495, SE = 0.113, *p* = 1.19 × 10^−5^) following Bonferroni correction to account for the number of regions tested. This region contains one of the genome-wide significant loci identified in the PGC BD2 study^44^, near *ZNF592*, a gene thought to play a role in cerebellar development. None of the other regions for any of the risk scores were significant, following correction for multiple testing. We therefore explored all regions reaching a threshold of *p* < 0.01 to discover if any of the nominally significant regions overlapped between the traits and diseases. Using this threshold, we identified 19 genomic regions for BD, 24 for total cholesterol, 25 for LDL, 25 for HDL, 21 for triglycerides, 25 for diabetes, 1 for presence of thyroid antibodies, and 4 for thyroid hormone levels (Fig. 4, Supplemental Table 11). Of the 144 regions identified, there were 121 unique regions, with 17 regions overlapping between traits.

The telomeric region on chromosome 4 (4p16.4) harbored local risk for both BD and diabetes that associated with mood disorder status in the pedigree (BD: β = 0.449, SE = 0.118, *p* = 1.4 × 10^−4^, diabetes: β = 0.323, SE = 0.114, *p* = 4.6 × 10^−3^). This region was previously identified as the region with the highest linkage with BD in this family (LOD 3.95)^21^. BD and diabetes local polygenic risk regions also overlapped on chromosome 7 (7p21.3), a region that contains the gene *NDUFA4* and has previously been associated with a psychosis phenotype^45^, and chromosome 17 (17p12), a region containing the genes *MYOCD*, *ARHGAP44*, and *ELAC2*, with no previous associations to mood disorders. BD and HDL local polygenic risk associated with mood disorder overlapped on chromosome 14 (14q12-13.1), a region harboring the gene *NPAS3*, which has previously been associated with schizophrenia susceptibility in linkage^46^ and association studies^47,48^. Diabetes, HDL, LDL, triglycerides, total cholesterol, and thyroid disorder local polygenic risk regions associated with mood disorder in this pedigree overlapped at multiple locations that were not additionally associated with polygenic bipolar local risk scores. The other regions were not found to overlap between traits, suggesting that the association of BD with risk for other traits in this pedigree is due to multiple pleiotropic regions spread across the genome.

## Discussion

Comorbidity of medical disease in individuals with psychiatric disorder is a major contributor to early mortality and severity of phenotype. Although previously attributed to poor lifestyle choices, recent genetic evidence suggests that pleiotropic loci that predispose to both mental illness and non-psychiatric disease may underlie at least some comorbidities. Here, we present a large multigenerational pedigree significantly enriched for bipolar and related mood disorders that we further genetically diagnose with both Mendelian disease and risk for complex phenotypes. In addition to identifying a unique pattern of co-segregating Mendelian disease variants associated with mood disorder status, polygenic risk profiling provides strong support for a shared genetic architecture between mood disorders and specific metabolic and endocrine traits.

The expectation in studies of complex disease in founder populations is that low-frequency disease-causing variants with strong effect sizes, that are rare in the general population but enriched or specific to the isolate, can be more easily identified compared to studies in outbred populations. However, previously published findings from the ASMAD population^21,22,29^ reveal a complex polygenic mode of inheritance, in line with findings from general population studies. Here, we corroborate this conclusion by reporting that BD polygenic risk profiling unequivocally supports a shared genetic architecture between BD disorder in the Amish and an outbred population of European ancestry. Furthermore, as expected from studies on the shared genetic etiology of psychiatric disorders^11^, PRS for major depressive disorder are also associated with BD in this pedigree. Interestingly, despite the high genetic correlation between BD and schizophrenia in the general population, the schizophrenia polygenic risk score was limited in its predictive ability in the ASMAD sample (*R*^2^ = 0.019). This may reflect an ascertainment bias in recruitment or a unique aspect of the phenotype in this extended pedigree. For example, out of 195 individuals with any reported psychiatric condition in the family, only a single individual presented with schizoaffective disorder and none with schizophrenia. Similarly, a study of major psychiatric disorders in mid-Western Amish in Ohio and Indiana reported lower rates of psychosis compared to other populations^49^.

Our previous analysis of regions of homozygosity did not reveal recessive risk variants for BD in this pedigree^21,22^. Conversely, here we report that PRS for BD and several metabolic traits are positively correlated with the inbreeding coefficient. Interestingly, we previously showed that Amish living in Lancaster County, Pennsylvania, where the ASMAD pedigree originates, have the longest homozygous-by-descent-regions^30^. Therefore, we suggest that reduced genetic diversity may underlie the accumulation of risk alleles for a range of diseases and traits.

Our identification of individuals who should present with a Mendelian disease, based on their carrier status of highly penetrant Mendelian variants, established a set of diseases that differ in frequency between those with and without mood disorder. Moreover, individuals carrying these variants in a manner insufficient for presentation of disease also demonstrated differences in mood disorder status. For example, a dominant familial hypercholesterolemia-associated APOB R3527Q variant, which has been previously identified as being carried by around 12% of Old Order Amish individuals^50^, was found in 19.7% of individuals with BPI, BPII or BP:NOS. This finding suggests that rather than one trait having a causal effect on the other (e.g., hypercholesterolemia “causing” BD) it is more likely that a gene variant, or the haplotype containing this Mendelian variant, convey risk for both disorders.

We identified risk for a number of complex metabolic and endocrinological diseases (lipid traits, diabetes, and clinical thyroid disease) that were significantly associated with mood disorder in this pedigree. The relationship between thyroid disorders and mood disorders has been acknowledged for many years^51^, with thyroid function associated with depressive symptoms, anxiety, and mania^52^. While metabolic syndrome in general^53,54^, and diabetes^55^ and lipid levels^56,57^ specifically, have been shown to be elevated in individuals with mood disorder, cross-trait analysis has not provided evidence for a significant genetic correlation between BD and any metabolic trait^5^. Our study on individual-level data in a large extended family reveals evidence for a genome-wide genetic correlation between mood disorder and specific metabolic traits. As this finding has not yet been replicated in an outbred population, we hypothesize that BD in the ASMAD family could represent a subtype of BD with high levels of metabolic and endocrinological disorders. This has been termed subgroup heterogeneity^58^, where a genetically distinct subset of individuals within a patient cohort is also genetically similar to individuals with another disease. There is already emerging evidence that such a subgroup exists for major depressive disorder^59^, and the identification of a similar subgroup in BD cohorts may help explain the epidemiological findings of associations between these disorders.

In an effort to quantify the regions that contribute to differences in risk scores, we analyzed risk scores for each approximately independent LD block and identified regions for which risk scores differ between individuals with mood disorder and their unaffected relatives. We identified a relatively small number of regions for each trait, covering 0.05–2.35% of the genome, that are associated with differences in risk scores at a *p*-value < 0.01. While 17 regions overlap between traits at this nominal cutoff, the majority of these regions are unique to each trait; out of the 19 regions identified as driving bipolar risk differences between affected and unaffected individuals, only four of these overlap with another trait. This suggests that, overall, regions that underlie the difference between affected and unaffected individuals polygenic risk for BD are not the same regions that underlie the difference between affected and unaffected individuals polygenic risk for metabolic traits.

We acknowledge several limitations within our current work that highlight avenues for future study. There are many cases of disease genes with different alleles leading to different phenotypes or a specific allele leading to a range of diverse phenotypes^60^. Although there are clear examples of homozygous variants causing a recessive Mendelian disease and a complex condition in carriers^60^, our study does not provide the resolution required to distinguish between a single gene vs. haplotype. Specifically, we cannot determine whether variation in the Mendelian disease gene alone, or variation at the level of the haplotype, is responsible for the shared genetic etiology of disease in this population. We expect that the long shared haplotypes observed in a genetic isolate are more likely to harbor multiple disease-contributing variants, and therefore produce the observed genetic pleiotropy. Furthermore, multiple mechanisms, such as genetic background or environmental effects may modify expressivity or penetrance of a specific allele^8^. While every attempt was made to limit our analyses to Mendelian variants predicted to cause disease, the presentation of Mendelian disease in members of the ASMAD pedigree could not be confirmed due to restrictions on re-contacting individuals in this legacy collection. As pleiotropic alleles continue to be identified, future studies would benefit from broadly phenotyping cases to fully capture the combination of traits and diseases present in each individual.

In conclusion, we demonstrate a case of genetic pleiotropy between a complex psychiatric disease with both Mendelian and complex metabolic and endocrine traits. We suggest this indicates a common genetic etiology for these traits in the extended Amish family. While our specific findings may not be extendable to other populations, we propose that each pedigree or population segregating psychiatric disorders will have unique combinations of additional medical traits. Taken together, our results denote that medical comorbidity between complex diseases and Mendelian disorders arises as a combination of chromosomal proximity of disease-causing variants and pleiotropy of disease genes. Elucidating these patterns could enhance the ability to identify regions and variants that contribute to disease in each unique population.

## Electronic supplementary material

Supplemental materials

Supplemental tables
