# Supplementary material for: Genetic pleiotropy between mood disorders, metabolic, and endocrine traits in a multigenerational pedigree
Source: Transl Psychiatry. 2018 Oct 12;8:218. doi: 10.1038/s41398-018-0226-3 (PMC6185949; doi:10.1038/s41398-018-0226-3)
Supplement: Supplementary file 1 — Supplemental materials [file 41398_2018_226_MOESM1_ESM.docx]

**Supplemental Materials for: Genetic pleiotropy between mood disorders, metabolic, and endocrine traits in a multigenerational pedigree**

Rachel L. Kember^1^, Liping Hou^2^, Xiao Ji^3^, Lars H. Andersen^4^, Arpita Ghorai^1^, Lisa N. Estrella^4^, Laura Almasy^1,5^, Francis J. McMahon^2^, Christopher Brown^1^, Maja Bućan^1,6^

1. Department of Genetics, Perelman School of Medicine, University of Pennsylvania, Philadelphia, PA 19104

2. Human Genetics Branch, National Institute of Mental Health Intramural Research Program, National Institutes of Health, Bethesda, MD 20892

3. Genomics and Computational Biology Program, Perelman School of Medicine, University of Pennsylvania, Philadelphia, PA 19104

4. Lancaster General Health/Penn Medicine, University of Pennsylvania Health System, Lancaster, PA 17602

5. Department of Biomedical and Health Informatics, Children’s Hospital of Philadelphia, Philadelphia, PA 19104

6. Department of Psychiatry, Perelman School of Medicine, University of Pennsylvania, Philadelphia, PA 19104

Corresponding author: Maja Bućan, bucan@upenn.edu

**Supplemental Methods**

**Sample**

The Amish Study of Major Affective Disorder consists of a large, extended bipolar disorder pedigree of 700 individuals with a small number of founders. Diagnoses of individuals were made following structured interviews (SADS-L) and a review of medical records by a psychiatric board using strict Research Diagnostic Criteria (RDC) and the Diagnostic and Statistical Manual of Mental Disorders, 4^th^ Edition (DSM-IV) for uniform clinical criteria ([1](#_ENREF_1)). The majority of affected individuals in the current pedigree are diagnosed as either BPI, BPII, or Major Depressive Disorder. Collection of blood samples followed diagnostic consensus, and lymphoblastoid cell lines were established by the Coriell Institute of Medical Research (CIMR). Signed informed consents were obtained, using language appropriate for Old Order Amish, to a) access medical records for the Amish Study clinicians exclusively to do diagnostic evaluations and clinical studies, and b) to perform collection of blood/tissue samples. In addition, all work contained within this study was approved by the IRB of the Perelman School of Medicine at the University of Pennsylvania.

**Phasing and Imputation**

Quality control of the raw genotype calls and imputed genotype calls was conducted using PLINK ([2](#_ENREF_2)). Individuals were excluded if they (1) had a call rate < 97% or (2) exhibited elevated levels of Mendelian inconsistencies. Genotyped variants were excluded from analysis if they: (1) had a call rate $<97\%$ or (2) had minor allele frequencies $<0.002\%$ (i.e., were singletons). Phasing was performed with SHAPEIT’s duoHMM option to account for known familial relationships, using the known ASMAD pedigree, with the genetic map from the HapMap phase II ([3](#_ENREF_3)), in 5Mb windows. Imputation was performed with IMPUTE2 in 5Mb windows with the following options: -use_prephased_g -known_haps_g $SAMPLE.haps -phase -buffer 500’. Following imputation, genotype dosages were converted to hard calls if above/below a threshold of 1.9/0.1, or otherwise set to missing. In total, 2,379,855 variants were imputed in 394 individuals. Quality control of the imputed calls included removing individuals who had a call rate < 97% (0 individuals), setting Mendelian errors to missing (124,668 variants), and removing variants with a call rate of <99% (120,611 variants) or had minor allele frequencies $<0.002\%$ (869,901 variants). 1,372,783 variants and 394 individuals passed QC. Imputation accuracy was assessed by comparing to whole genome sequence data available for a subset of family members (99.3% concordance).

**Whole genome sequencing**

Paired-end reads of length 70 bp (35 bp at each end) were mapped to the National Center for Biotechnology Information (NCBI) human reference genome (build 37.2) using a Bayesian mapping pipeline ([4](#_ENREF_4)). Variant calls were performed by CGI using version 2.0.3.1 of their pipeline. False discovery rate estimates for SNP calls of the CGI platform are 0.2–0.6% ([5](#_ENREF_5)). Gene annotations were based on the NCBI build 37.2 seq_gene file contained in a NCBI annotation build. The variant calls within the WGS were processed using the cgatools software (version 1.5.0, build 31) made available by CGI. The listvar tool was used to generate a master list of the 11.1 M variants present in the 80 Amish samples. The testvar tool was used to determine presence and absence of each variant within the 80 Amish WGS. Only variants with high variant call scores (“VQHIGH” tag in the data files) were included.

**Human disease catalog**

The Human Genome Mutation Database (HGMD) catalogs known disease associated variants (<http://www.hgmd.org/>; ([6](#_ENREF_6))). Most of the clinical phenotypes in the database are monogenic diseases. In the June 2013 release it contained 141,000 different variants in ~5,700 genes (“HGMD disease genes”). We examined all variants present in ASMAD in 3456 HGMD disease genes (‘DM’ tag in HGMD).

**Curation of HGMD disease causing variants**

251 variants present in ASMAD were annotated as being a ‘disease mutation’ in HGMD. In order to further refine this list to identify true disease causing mutations, we first removed all variants found to be present at >1% frequency in 1000 Genomes ([7](#_ENREF_7)) or ExAC ([8](#_ENREF_8)), assuming that disease causing alleles will be rare in a population (total remaining variants=154). Next, we merged this list with annotation from ClinVar ([9](#_ENREF_9)) and selected all variants annotated as pathogenic (n=62). We then identified whether each variant caused disease in a recessive or dominant model, and used this information to identify individuals in the ASMAD family predicted to display the disease phenotype based on their allelic status (n=25 variants). For these 25 variants, we expanded upon the HGMD and ClinVar annotation by applying criteria recommended by the American College of Medical Genetics and Genomics (ACMG; ([10](#_ENREF_10))) for the interpretation of sequence variants, and classified the variants into the categories “pathogenic”, “likely pathogenic”, “uncertain significance”, “likely benign” and “benign”. Out of the 25, ACMG criteria classified 3 variants as “benign”, 6 variants as “uncertain significance”, 10 variants as “likely pathogenic” and 6 variants as “pathogenic”. For analyses we removed the 3 variants classified as benign and retained the others (n=22 variants).

**Loss of function variants**

Variants were annotated using VEP LOFTEE ([11](#_ENREF_11)). We detect 1177 putative protein truncating (frameshift, splice donor, splice acceptor, and stop-gained) variants. Using filters supplied by LOFTEE, we removed 176 variants (see tables below), resulting in 1001 high confidence loss of function variants (HC-LoF).

| Filter | Description | Number of variants |
| --- | --- | --- |
| END_TRUNC | Variant falls in the last 5% of the transcript | 28 |
| EXON_INTRON_UNDEF | Exon or intron boundaries undefined for this transcript | 1 |
| NON_CAN_SPLICE | Variant falls in non-canonical splice site | 3 |
| NON_CAN_SPLICE_SURR | Variant falls in exon with non-canonical splice site | 7 |

| Flag | Description | Number of variants |
| --- | --- | --- |
| NAGNAG_SITE | Splice acceptor has in-frame AG acceptor site one codon away | 24 |
| SINGLE_EXON | Variant falls in a single exon transcript | 113 |

We identified 167 HC-LoF variants in disease genes, of which 71 are in a homozygous state in one or more individuals. There are 83 rare (<1% in ExAC and 1000G) HC-LoF in disease genes, 17 of which are found in a homozygous state in one or more individuals.

**EMMAX and MONSTER**

A Balding-Nichols kinship matrix was constructed from the imputed whole genome sequence data following removal of all variants with >5% missing and <1% allele frequency, using the command emmax-kin in the EMMAX package. Association analysis for all variants was performed using EMMAX (Version from February 2012, ([12](#_ENREF_12))), a statistical test for association analysis using mixed models that accounts for the population structure within the sample. Gene-based association tests were performed using MONSTER ([13](#_ENREF_13)), a statistical test that generalizes the SKAT-O method and uses a mixed effects model to account for population structure. The kinship matrix used in the EMMAX analysis was also used in the MONSTER analysis.

**Association rule discovery**

Association rules for the comorbidity of mood disorder and Mendelian diseases in individuals within the ASMAD pedigree were determined using the apriori algorithm from the arules package in R ([14](#_ENREF_14)). An itemset was created for each individual, consisting of the affected status for mood disorder (“unaffected” or “broad extended” phenotype) and any comorbid Mendelian disease as determined by the allelic status of the disease causing variant in that individual. Association rules for the frequent itemsets were generated using the apriori command. Rules were then limited to those with either “broad extended” or “unaffected” on the left hand side of the rule (antecedents) and Mendelian diseases on the right hand side of the rule (consequents).

**Polygenic risk scores**

A polygenic risk score is generated for each individual as the sum of all variants they carry, weighted by the effect that variant has on phenotype. Polygenic risk scores were generated using the PRSice package ([15](#_ENREF_15)), with multiple GWAS summary statistics as the base dataset (see Supplemental table 9), and imputed whole genome sequence data in ASMAD as the target dataset. As recommended in the software, we performed p-value informed clumping on the genotype data with an r2 = 0.1 and a distance threshold of 250kb, following exclusion of the MHC region. The optimal p-value threshold for PRS was defined as that which explained the most phenotypic variation for mood disorder in the ASMAD pedigree, out of a set of pre-determined thresholds (p ≤ 0.0001, 0.0005, 0.001, 0.005, 0.01, 0.05, 0.1, 0.5). For traits identified as significantly associated with mood disorder in this pedigree, we show barplots indicating the model fit and p-value for association at each p-value threshold tested (Supplemental figure 29).

**Statistical analysis**

Polygenic risk scores for the optimal p-value threshold for each trait were standardized mean=0 and standard deviation=1. Linear mixed model analyses were selected to model outcomes (Narrow phenotype, Broad phenotype, Broad extended phenotype, Depression phenotype) while accounting for relatedness. The analysis was performed using the pedigreemm package in R ([16](#_ENREF_16)), with PRS as the independent variable and phenotype as the dependent variable. An empirical kinship matrix, constructed from the genome-wide SNP data, by the Balding-Nicols method using rvtests ([17](#_ENREF_17)) was fitted as a random effect to account for relatedness between individuals. Risk scores were evaluated for 22 traits, and so a bonferroni corrected p-value<0.0023 (0.05/22) was selected as the level for statistical significance.

**Polygenic transmission disequilibrium**

A test for polygenic transmission disequilibrium has recently been described by Weiner et al. ([18](#_ENREF_18)). We modified this test to allow the comparison of multiple affected and unaffected siblings. We selected all nuclear families with at least one child with Broad phenotype (BPI, BPII, BP:NOS, MDDR, number of families=46). For each family, the polygenic transmission disequilibrium deviation was calculated as follows:

$$pTDTdeviation=\frac{Average PRS affected-Average PRS unaffected}{SD(PRS all siblings)}$$

The test statistic for pTDT deviation was then calculated based on all families as follows:

$$tpTDT=\frac{Mean(pTDT deviation)}{SD(pTDT deviation)/\surd n}$$

Where n is the number of families.

**Local polygenic risk score**

Drawing on work from Shi et al. ([19](#_ENREF_19)), on local genetic correlation, we developed a method for establishing local genetic risk, i.e. genetic risk based on specific regions of the genome. First, we apportioned variants and their corresponding GWAS summary statistics into approximately independent LD blocks ([20](#_ENREF_20)). Polygenic risk scores were then generated for each individual in the pedigree based on just the variants within each LD block. For each disease or trait the p-value cut-off for local genetic risk was based on that which explained the most phenotypic variation for mood disorder in the ASMAD pedigree in the genome-wide PRS (calculated as described above). Local risk scores were standardized mean=0 and standard deviation=1. As above, the association of local risk scores with presence of mood disorder were calculated using a linear mixed model with an empirical kinship matrix (constructed from the genome-wide SNP data) fitted as a random effect to account for relatedness between individuals.

**Supplemental Tables and Figures**

**Supplemental Table 1**: Available known phenotypes and co-morbidities for ASMAD population. Around one third of individuals are affected by psychiatric disorder. We identified additional disease co-morbidities using sequence analysis.

|  | **Phenotype Model** | | | | | **Co-morbid** | | |
| --- | --- | --- | --- | --- | --- | --- | --- | --- |
|  | BPI | Narrow | Broad | Depression | Broad Extended | Anxiety | Psychosis | Postpartum |
| BPI | 78 | 78 | 78 |  | 78 |  | 37 | 1 |
| BPII |  | 13 | 13 |  | 13 |  | 1 | 1 |
| BP NOS |  | 20 | 20 |  | 20 |  | 3 |  |
| MDDR |  |  | 20 | 20 | 20 | 1 | 2 | 2 |
| MDD |  |  |  | 13 | 13 |  |  |  |
| Minor Depression |  |  |  | 30 | 30 | 3 |  | 3 |
| Other |  |  |  |  | 23 | 3 | 2 | 1 |
| Total | 78 | 111 | 131 | 63 | 195 | 7 | 45 | 8 |
| Percentage | 13.4% | 19.0% | 22.5% | 10.8% | 33.4% | 1.2% | 7.7% | 1.4% |
| Mean age onset | 21.1 | 21.4 | 22.1 | 26.4 | 22.6 |  |  |  |
| Standard deviation | ±6.2 | ±6.3 | ±7.5 | ±12.6 | ±8.7 |  |  |  |

**Supplemental Table 2**: Genes with damaging variants that are rare in 1000 Genomes and ExAC (<2%) and common in ASMAD (>5%) (see excel file)

**Supplemental Figure 1**: Q-Q plot for results of MONSTER gene-based association test. No genes reaching genome-wide significance were found to be associated with mood disorder in this pedigree.


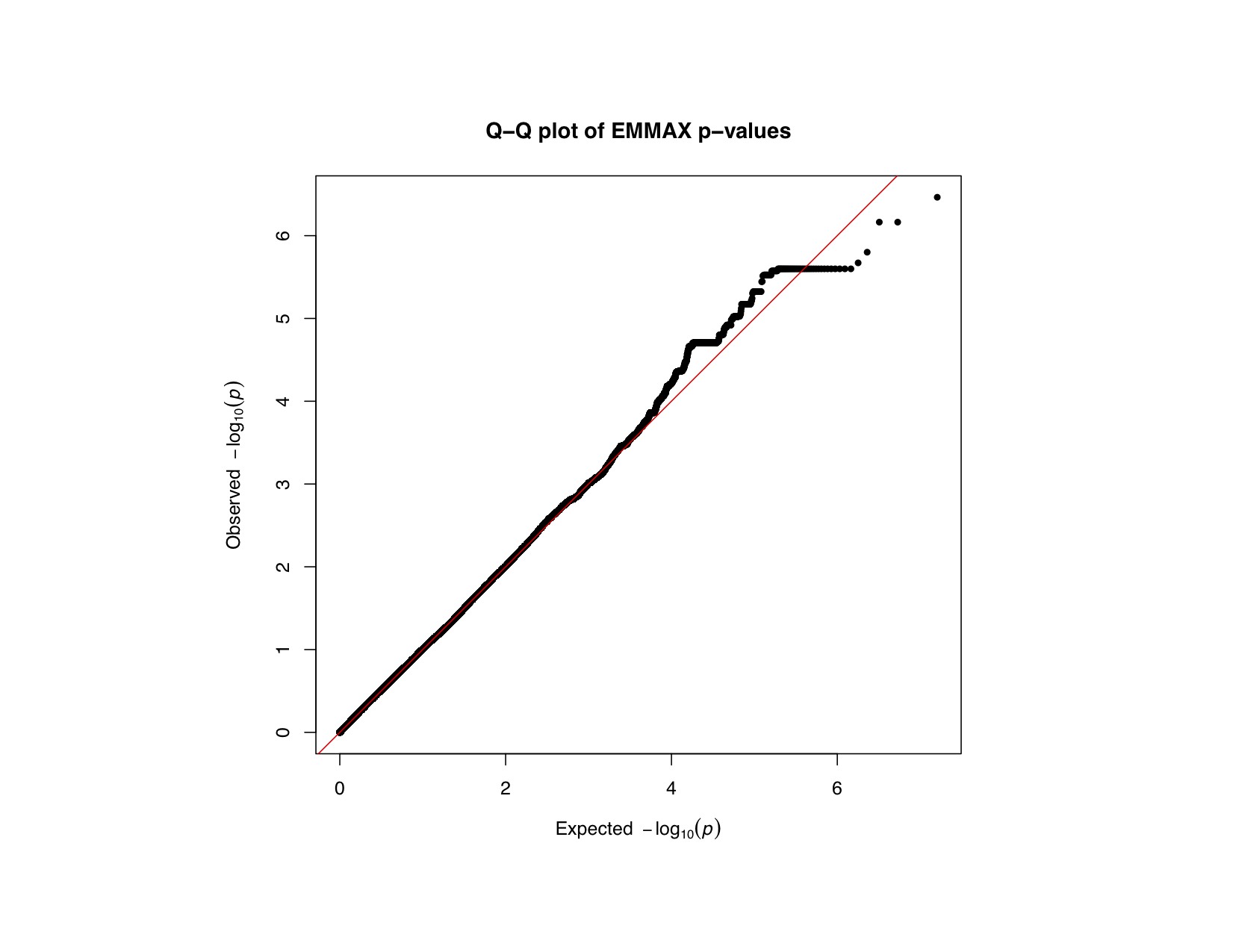


**Supplemental Figure 2**: Q-Q plot for results of EMMAX variant-based association test. No variants reaching genome-wide significance were found to be associated with mood disorder in this pedigree.

**Supplemental Table 3**: MONSTER results for genes with p-value <0.001

| Gene | p-value | Chr | CDS_Begin | CDS_End | HGMD | OMIM_disorder |
| --- | --- | --- | --- | --- | --- | --- |
| *TNN* | 2.00E-04 | 1 | 179390717 | 179672150 | . | . |
| *IGLL1* | 2.14E-04 | 22 | 23915313 | 23922495 | DM | Agammaglobulinemia 2 |
| *TMPRSS4* | 3.37E-04 | 11 | 117947727 | 117990556 | DM | . |
| *GALNS* | 3.93E-04 | 16 | 88880142 | 88923374 | DM | Mucopolysaccharidosis IVA |
| *T* | 6.23E-04 | 6 | 166571146 | 166582157 | DM | Neural tube defects, susceptibility to; Sacral agenesis with vertebral anomalies |
| *TCN2* | 7.13E-04 | 22 | 31003070 | 31023047 | DM | Transcobalamin II deficiency |

**Supplemental Table 4**: EMMAX results for nonsynonymous exonic variants with p-value<0.001

| Gene | Chr | Start | Ref | Alt | snp138 | HGMD | P-value |
| --- | --- | --- | --- | --- | --- | --- | --- |
| *IGLL1* | 22 | 23917192 | G | T | rs116041505 | DM | 6.74E-06 |
| *TNN* | 1 | 175046789 | A | G | rs2072032 |  | 6.56E-05 |
| *TCN2* | 22 | 31006860 | A | G | rs9606756 | DM | 7.06E-05 |
| *ATG2B* | 14 | 96761321 | A | G | rs149239345 |  | 0.000137401 |
| *ADAMTSL3* | 15 | 84706461 | C | T | rs950169 | DM | 0.000234225 |
| *RNF224* | 9 | 140123132 | C | G | rs6606565 |  | 0.00025956 |
| *GRM7* | 3 | 7340413 | G | A | rs372059810 | DM | 0.000363749 |
| *SNIP1* | 1 | 38003443 | T | C |  |  | 0.000387613 |
| *DEFB125* | 20 | 76978 | G | A | rs140898692 |  | 0.000417261 |
| *CES5A* | 16 | 55880480 | A | C | rs11860456 |  | 0.000495823 |
| *COL4A3* | 2 | 228128568 | G | A | rs34505188 | DM | 0.000764593 |
| *COL4A3* | 2 | 228131169 | A | G | rs11677877 | DM | 0.000764593 |
| *LOXHD1* | 18 | 44137400 | C | T | rs118174674 | DM | 0.000771686 |
| *GALNS* | 16 | 88891240 | C | A | rs2303269 | DM | 0.00080526 |
| *KCNAB1* | 3 | 155991410 | C | G | rs13093003 |  | 0.000810128 |
| *CHRNA5* | 15 | 78882925 | G | A | rs16969968 |  | 0.000955548 |
| *FBXW12* | 3 | 48419898 | G | A | rs79525977 |  | 0.00096863 |


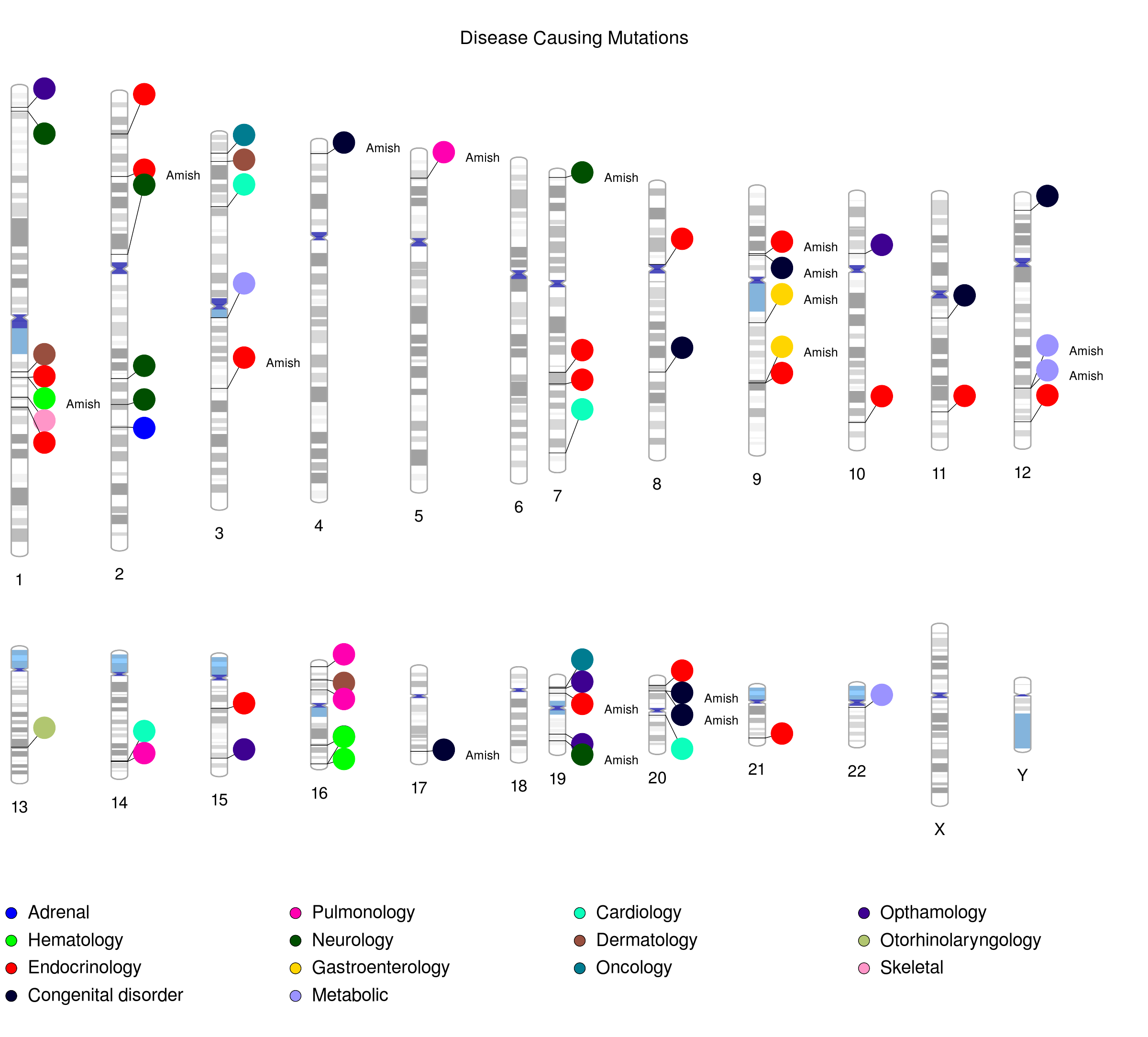


**Supplemental Figure 3**: Many disease causing mutations are found in the ASMAD pedigree. Location and type of disease causing mutation found within the ASMAD pedigree. 62 disease-causing mutations were identified within the pedigree, of which 17 mutations are known Amish disease alleles ([21-23](#_ENREF_21)).

**Supplemental Table 5**: High confidence disease causing mutations carried in the ASMAD pedigree (see excel file)

**Supplemental Table 6**: Disease causing mutations predicted to cause disease in the ASMAD pedigree (see excel file)

**Supplemental figures 4-21 (below)**: Nuclear family graph for ASMAD. Each node is a nuclear family. An arrow from one family to another means that the child of the first family becomes in parent of the second family. Families in red contain individuals with the disease (noted in the header) according to their genetic data.

**Supplemental Figure 4**: Ocular skeletal abnormalities

**Supplemental Figure 5**: Obstructive lung disease

**Supplemental Figure 6**: Trimethylaminuria

**Supplemental Figure 7**: Idiopathic epilepsy

**Supplemental Figure 8**: Harderoporphyria

**Supplemental Figure 9**: Xerocytosis

**Supplemental Figure 10**: Corneal dystrophy

**Supplemental Figure 11**: Small fibre neuropathy

**Supplemental Figure 12**: Bronchiectasis

**Supplemental Figure 13**: Bardet-Biedl

**Supplemental Figure 14**: Glaucoma

**Supplemental Figure 15**: Apolipoprotein_C-III

**Supplemental Figure 16**: McKusick-Kaufman

**Supplemental Figure 17**: Hypogonadotropic_hypogonadism

**Supplemental Figure 18**: Long QT syndrome

**Supplemental Figure 19**: Hyperprolinemia

**Supplemental Figure 20**: Familial hypercholesterolemia

**Supplemental Figure 21**: Adrenocorticol_hyperplasia

**Supplemental Figure 22**: Percentage of individuals with multiple lipid variants versus the expected percentage of individuals.

**Supplemental Table 7**: Heritability of phenotype models within ASMAD

| Trait | # individuals | H2r | P-value |
| --- | --- | --- | --- |
| BPI | 75 | 0.33 | 0.003 |
| Narrow (BPI, BPII, BP:NOS) | 90 | 0.45 | 0.0001 |
| Broad (BPI, BPII, BP:NOS, MDDR) | 120 | 0.52 | 0.00001 |
| Broad extended (Any psychiatric phenotype) | 203 | 0.81 | 9.76E-10 |
| Depression (MDD, MDDR, Minor Depression) | 63 | 0.72 | 0.000005 |
| Well (Unaffected) | 398 | 0.8 | 9.76E-10 |

**Supplemental Table 8**: Association of polygenic risk scores for each phenotype model in ASMAD. P-values, effect sizes and standard errors were generated using linear mixed models to account for relatedness (see excel sheet).

**Supplemental Figure 23**: Bipolar disorder risk score by phenotype. Risk score is higher in affected individuals, particularly those with BPI, BPII, BP:NOS, and MDDR.

**Supplemental Figure 24**: Bipolar disorder risk score by parents phenotype. Individuals with two affected parents have higher risk scores than those with one affected parent. Individuals with two unaffected parents have lower risk scores.

**Supplemental Figure 25**: Bipolar disorder risk scores by lineage. Individuals who can be traced back to the pioneer members of this pedigree have a higher polygenic risk score than Amish married-in individuals.

**Supplemental Figure 26**: Bipolar disorder risk scores by nuclear family. There is an extensive range of risk scores across the pedigree, with members of the same nuclear family being similar to each other regardless of phenotype.

**Supplemental Figure 27**: Bipolar disorder risk score is significantly correlated with inbreeding, as measured by PLINKs F value.

**Supplemental Figure 28**: Correlation between polygenic risk scores for bipolar disorder and (from top left) HDL, LDL, triglycerides, total cholesterol.

**Supplemental Table 9**: GWAS studies and consortia from which summary statistics were collected. Code for converting all summary statistics to input format required by PRSice is available upon request.

| **Category** | **Trait/GWAS** | **Reference** | **Study/Consortium** | **Number of SNPs** |
| --- | --- | --- | --- | --- |
| Psychiatric Disorder | Bipolar Disorder (PGC BD2) | ([24](#_ENREF_24)) | Psychiatric Genomics Consortium | ~13.4 million |
|  | Major Depression (PGC MDD) | Unpublished data |  | ~12.9 million |
|  | Schizophrenia (PGC SCZ2) | ([25](#_ENREF_25)) |  | ~9.4 million |
|  | Autism (PGC AUT) | ([26](#_ENREF_26)) |  | ~9.5 million |
|  | Cross disorder (PGC) | ([27](#_ENREF_27)) |  | ~1.2 million |
|  | ANGST Case-control | ([28](#_ENREF_28)) | Anxiety Neuro Genetics Study | ~6.3 million |
|  | ANGST Quantitative phenotype |  |  | ~6.3 million |
|  | ADHD (EAGLE) | ([29](#_ENREF_29)) | The EArly Genetics and Lifecourse Epidemiology Consortium | ~5.8 million |
| Personality | Neuroticism (GPC2) | ([30](#_ENREF_30)) | Genetics of Personality Consortium | ~6.9 million |
|  | Extraversion (GPC2) | ([31](#_ENREF_31)) |  | ~6.9 million |
| Educational attainment | SSGAC (EA 2016) | ([32](#_ENREF_32)) | Social Science Genetic Association Consortium | ~8.1 million |
| Alzheimer’s | IGAP Stage 1 | ([33](#_ENREF_33)) | International Genomics of Alzheimer's Project | ~7 million |
| Diabetes | GoT2D (DIAGRAM) | ([34](#_ENREF_34)) | DIAbetes Genetics Replication And Meta-analysis | ~2.9 million |
| Thyroid | TPOAb serum levels | ([35](#_ENREF_35)) | Medici et al., 2014 | ~2.7 million |
|  | TPOAb case control |  |  | ~2.7 million |
| Lipids | HDL (Global Lipids) | ([36](#_ENREF_36)) | Global Lipids | ~2.5 million |
|  | LDL (Global Lipids) |  |  | ~2.5 million |
|  | TC (Global Lipids) |  |  | ~2.5 million |
|  | TG (Global Lipids) |  |  | ~2.5 million |
| Cardiovascular Disease | CAD additive | ([37](#_ENREF_37)) | CARDIoGRAMplusC4D | ~9.5 million |
|  | CAD recessive |  |  | ~6.9 million |
|  | MI additive |  |  | ~9.2 million |

**Supplemental Table 10**: Leave-one-out analysis of risk score association with BD following removal of each set of individuals with predicted Mendelian disease (see excel sheet)

**Supplemental Table 11**: Genomic regions with significantly different risk scores between affected and unaffected individuals (see excel sheet)

**Supplemental Figure 29**: Model fit and p-value for association with mood disorder at each p-value threshold tested (p ≤ 0.0001, 0.0005, 0.001, 0.005, 0.01, 0.05, 0.1, 0.5) for (left to right) Bipolar disorder PRS, Diabetes PRS, HDL PRS, LDL PRS, Triglycerides PRS, Total Cholesterol PRS, TPOAb case control PRS, TPOAb levels PRS.

**References**

1. Egeland JA, Sussex JN, Endicott J, Hostetter AM, Offord DR, Schwab JJ, et al. The Impact of Diagnoses on Genetic Linkage Study for Bipolar Affective Disorders among the Amish. Psychiatric Genetics. 1990;1(2):5&hyhen;18.

2. Purcell S, Neale B, Todd-Brown K, Thomas L, Ferreira MA, Bender D, et al. PLINK: a tool set for whole-genome association and population-based linkage analyses. American journal of human genetics. 2007;81(3):559-75. Epub 2007/08/19.

3. Frazer KA, Ballinger DG, Cox DR, Hinds DA, Stuve LL, Gibbs RA, et al. A second generation human haplotype map of over 3.1 million SNPs. Nature. 2007;449(7164):851-61. Epub 2007/10/19.

4. Carnevali P, Baccash J, Halpern AL, Nazarenko I, Nilsen GB, Pant KP, et al. Computational techniques for human genome resequencing using mated gapped reads. Journal of computational biology : a journal of computational molecular cell biology. 2012;19(3):279-92. Epub 2011/12/20.

5. Drmanac R, Sparks AB, Callow MJ, Halpern AL, Burns NL, Kermani BG, et al. Human genome sequencing using unchained base reads on self-assembling DNA nanoarrays. Science. 2010;327(5961):78-81. Epub 2009/11/07.

6. Stenson PD, Mort M, Ball EV, Shaw K, Phillips A, Cooper DN. The Human Gene Mutation Database: building a comprehensive mutation repository for clinical and molecular genetics, diagnostic testing and personalized genomic medicine. Human genetics. 2014;133(1):1-9. Epub 2013/10/01.

7. Auton A, Brooks LD, Durbin RM, Garrison EP, Kang HM, Korbel JO, et al. A global reference for human genetic variation. Nature. 2015;526(7571):68-74. Epub 2015/10/04.

8. Lek M, Karczewski KJ, Minikel EV, Samocha KE, Banks E, Fennell T, et al. Analysis of protein-coding genetic variation in 60,706 humans. Nature. 2016;536(7616):285-91. Epub 2016/08/19.

9. Landrum MJ, Lee JM, Benson M, Brown G, Chao C, Chitipiralla S, et al. ClinVar: public archive of interpretations of clinically relevant variants. Nucleic acids research. 2016;44(D1):D862-8. Epub 2015/11/20.

10. Richards S, Aziz N, Bale S, Bick D, Das S, Gastier-Foster J, et al. Standards and guidelines for the interpretation of sequence variants: a joint consensus recommendation of the American College of Medical Genetics and Genomics and the Association for Molecular Pathology. Genetics in medicine : official journal of the American College of Medical Genetics. 2015;17(5):405-24. Epub 2015/03/06.

11. McLaren W, Gil L, Hunt SE, Riat HS, Ritchie GR, Thormann A, et al. The Ensembl Variant Effect Predictor. Genome biology. 2016;17(1):122. Epub 2016/06/09.

12. Kang HM, Sul JH, Service SK, Zaitlen NA, Kong SY, Freimer NB, et al. Variance component model to account for sample structure in genome-wide association studies. Nature genetics. 2010;42(4):348-54. Epub 2010/03/09.

13. Jiang D, McPeek MS. Robust rare variant association testing for quantitative traits in samples with related individuals. Genetic epidemiology. 2014;38(1):10-20. Epub 2013/11/20.

14. Hahsler M, Grün B, Hornik K. arules - A Computational Environment for Mining Association Rules and Frequent Item Sets. 2005. 2005;14(15):25. Epub 2005-01-31.

15. Euesden J, Lewis CM, O'Reilly PF. PRSice: Polygenic Risk Score software. Bioinformatics. 2015;31(9):1466-8. Epub 2015/01/01.

16. Vazquez AI, Bates DM, Rosa GJ, Gianola D, Weigel KA. Technical note: an R package for fitting generalized linear mixed models in animal breeding. Journal of animal science. 2010;88(2):497-504. Epub 2009/10/13.

17. Zhan X, Hu Y, Li B, Abecasis GR, Liu DJ. RVTESTS: an efficient and comprehensive tool for rare variant association analysis using sequence data. Bioinformatics. 2016;32(9):1423-6. Epub 2016/05/08.

18. Weiner DJ, Wigdor EM, Ripke S, Walters RK, Kosmicki JA, Grove J, et al. Polygenic transmission disequilibrium confirms that common and rare variation act additively to create risk for autism spectrum disorders. Nature genetics. 2017;49(7):978-85. Epub 2017/05/16.

19. Shi H, Mancuso N, Spendlove S, Pasaniuc B. Local genetic correlation gives insights into the shared genetic architecture of complex traits. bioRxiv. 2016.

20. Berisa T, Pickrell JK. Approximately independent linkage disequilibrium blocks in human populations. Bioinformatics. 2016;32(2):283-5. Epub 2015/09/24.

21. Puffenberger EG, Jinks RN, Sougnez C, Cibulskis K, Willert RA, Achilly NP, et al. Genetic mapping and exome sequencing identify variants associated with five novel diseases. PloS one. 2012;7(1):e28936. Epub 2012/01/27.

22. Strauss KA, Puffenberger EG. Genetics, medicine, and the Plain people. Annual review of genomics and human genetics. 2009;10:513-36. Epub 2009/07/28.

23. Strauss KA, Puffenberger EG, Morton DH. One community's effort to control genetic disease. American journal of public health. 2012;102(7):1300-6. Epub 2012/05/19.

24. Stahl E, Forstner A, McQuillin A, Ripke S, Ophoff R, Scott L, et al. Genomewide association study identifies 30 loci associated with bipolar disorder. bioRxiv. 2017.

25. Schizophrenia Working Group of the Psychiatric Genomics Consortium. Biological insights from 108 schizophrenia-associated genetic loci. Nature. 2014;511(7510):421-7. Epub 2014/07/25.

26. The Autism Spectrum Disorders Working Group of The Psychiatric Genomics Consortium. Meta-analysis of GWAS of over 16,000 individuals with autism spectrum disorder highlights a novel locus at 10q24.32 and a significant overlap with schizophrenia. Molecular autism. 2017;8:21. Epub 2017/05/26.

27. Cross-Disorder Group of the Psychiatric Genomics Consortium. Identification of risk loci with shared effects on five major psychiatric disorders: a genome-wide analysis. Lancet. 2013;381(9875):1371-9. Epub 2013/03/05.

28. Otowa T, Hek K, Lee M, Byrne EM, Mirza SS, Nivard MG, et al. Meta-analysis of genome-wide association studies of anxiety disorders. Molecular psychiatry. 2016;21(10):1391-9. Epub 2016/01/13.

29. Middeldorp CM, Hammerschlag AR, Ouwens KG, Groen-Blokhuis MM, Pourcain BS, Greven CU, et al. A Genome-Wide Association Meta-Analysis of Attention-Deficit/Hyperactivity Disorder Symptoms in Population-Based Pediatric Cohorts. Journal of the American Academy of Child and Adolescent Psychiatry. 2016;55(10):896-905 e6. Epub 2016/09/25.

30. de Moor MH, van den Berg SM, Verweij KJ, Krueger RF, Luciano M, Arias Vasquez A, et al. Meta-analysis of Genome-wide Association Studies for Neuroticism, and the Polygenic Association With Major Depressive Disorder. JAMA psychiatry. 2015;72(7):642-50. Epub 2015/05/21.

31. van den Berg SM, de Moor MH, Verweij KJ, Krueger RF, Luciano M, Arias Vasquez A, et al. Meta-analysis of Genome-Wide Association Studies for Extraversion: Findings from the Genetics of Personality Consortium. Behavior genetics. 2016;46(2):170-82. Epub 2015/09/13.

32. Okbay A, Beauchamp JP, Fontana MA, Lee JJ, Pers TH, Rietveld CA, et al. Genome-wide association study identifies 74 loci associated with educational attainment. Nature. 2016;533(7604):539-42. Epub 2016/05/27.

33. Lambert JC, Ibrahim-Verbaas CA, Harold D, Naj AC, Sims R, Bellenguez C, et al. Meta-analysis of 74,046 individuals identifies 11 new susceptibility loci for Alzheimer's disease. Nature genetics. 2013;45(12):1452-8. Epub 2013/10/29.

34. Mahajan A, Go MJ, Zhang W, Below JE, Gaulton KJ, Ferreira T, et al. Genome-wide trans-ancestry meta-analysis provides insight into the genetic architecture of type 2 diabetes susceptibility. Nature genetics. 2014;46(3):234-44. Epub 2014/02/11.

35. Medici M, Porcu E, Pistis G, Teumer A, Brown SJ, Jensen RA, et al. Identification of novel genetic Loci associated with thyroid peroxidase antibodies and clinical thyroid disease. PLoS genetics. 2014;10(2):e1004123. Epub 2014/03/04.

36. Willer CJ, Schmidt EM, Sengupta S, Peloso GM, Gustafsson S, Kanoni S, et al. Discovery and refinement of loci associated with lipid levels. Nature genetics. 2013;45(11):1274-83. Epub 2013/10/08.

37. Nikpay M, Goel A, Won HH, Hall LM, Willenborg C, Kanoni S, et al. A comprehensive 1,000 Genomes-based genome-wide association meta-analysis of coronary artery disease. Nature genetics. 2015;47(10):1121-30. Epub 2015/09/08.
